# Prebiotic galactooligosaccharide improves piglet growth performance and intestinal health associated with alterations of the hindgut microbiota during the peri-weaning period

**DOI:** 10.1186/s40104-024-01047-y

**Published:** 2024-06-13

**Authors:** Timothy E. Boston, Feng Wang, Xi Lin, Sung Woo Kim, Vivek Fellner, Mark F. Scott, Amanda L. Ziegler, Laurianne Van Landeghem, Anthony T. Blikslager, Jack Odle

**Affiliations:** 1 Department of Animal Science, College of Ag and Life Sciences, North Carolina State University, Raleigh, NC 27695 USA; 2 Department of Clinical Sciences, North Carolina State University, Raleigh, NC 27695 USA; 3 Department of Molecular Biomedical Sciences, College of Veterinary Medicine, North Carolina State University, Raleigh, NC 27695 USA; 4 Milk Specialties Global, Eden Prairie, MN 55344 USA

**Keywords:** Galactooligosaccharide, Gut microbiome, Intestinal villi, Prebiotics, Weaning

## Abstract

**Background:**

Weaning stress reduces growth performance and health of young pigs due in part to an abrupt change in diets from highly digestible milk to fibrous plant-based feedstuffs. This study investigated whether dietary galactooligosaccharide (GOS), supplemented both pre- and post-weaning, could improve growth performance and intestinal health via alterations in the hindgut microbial community.

**Methods:**

Using a 3 × 2 factorial design, during farrowing 288 piglets from 24 litters received either no creep feed (FC), creep without GOS (FG–) or creep with 5% GOS (FG+) followed by a phase 1 nursery diet without (NG–) or with 3.8% GOS (NG+). Pigs were sampled pre- (D22) and post-weaning (D31) to assess intestinal measures.

**Results:**

Creep fed pigs grew 19% faster than controls (*P* < 0.01) prior to weaning, and by the end of the nursery phase (D58), pigs fed GOS pre-farrowing (FG+) were 1.85 kg heavier than controls (*P* < 0.05). Furthermore, pigs fed GOS in phase 1 of the nursery grew 34% faster (*P* < 0.04), with greater feed intake and efficiency. Cecal microbial communities clustered distinctly in pre- vs. post-weaned pigs, based on principal coordinate analysis (*P* < 0.01). No effects of GOS were detected pre-weaning, but gruel creep feeding increased Chao1 α-diversity and altered several genera in the cecal microbiota (*P* < 0.05). Post-weaning, GOS supplementation increased some genera such as *Fusicatenibacter* and *Collinsella,* whereas others decreased such as *Campylobacter* and *Frisingicoccus* (*P* < 0.05). Changes were accompanied by higher molar proportions of butyrate in the cecum of GOS-fed pigs (*P* < 0.05).

**Conclusions:**

Gruel creep feeding effectively improves suckling pig growth regardless of GOS treatment. When supplemented post-weaning, prebiotic GOS improves piglet growth performance associated with changes in hindgut microbial composition.

**Supplementary Information:**

The online version contains supplementary material available at 10.1186/s40104-024-01047-y.

## Background

The nursery period in swine production is challenged with high rates of morbidity and mortality stemming from stark reductions in feed intake owing to abrupt changes in diet composition, socio-hierarchical challenges, and overall weaning stress. Dietary antibiotics have historically been utilized post-weaning due to their ability to decrease pathogenic bacteria. However, there are growing concerns of antimicrobial resistant bacteria due to the excess use of antibiotics in swine feeds [1]. To mitigate the negative impacts of weaning and shift away from antibiotics, the use of creep feeding during farrowing and the supplementation of prebiotics during the farrowing and nursery periods have been explored as potential solutions.

Supplementing sow’s milk with creep feed to suckling piglets is a practice that has been employed for many years and has several benefits such as increased weaning weight, feed familiarity, and post-weaning feed intake [2–4]. Whereas some studies report that intake of dry creep feed may be negligible [5], there are several innovations such as liquid gruel creep diets that increase intakes of creep feed [6, 7].

Prebiotics are nondigestible feed ingredients that promote the growth of beneficial microorganisms in the gut. Different prebiotics range from mannan-oligosaccharides, fructo-oligosaccharides, to galactooligosaccharides (GOS). They are commonly introduced as a supplement to swine nursery diets where their fermentation may increase short chain fatty acid (SCFA) production, shift microbial communities and impact immunologic and metabolic processes in the GI tract [8–11]. Whereas prebiotics were originally seen as a replacement of cellulose for a more digestible fiber source [12], we now have sequencing technologies to examine detailed gut microbial changes induced by prebiotic inclusion in diets [13]. Given that galactose is a natural constituent of milk, GOS may be particularly well-suited for use in early life. Indeed, commercial GOS products are generally recognized as safe and have been used as prebiotics in formulas for infants [14]. GOS is synthesized by incubating high concentrations of lactose with beta-galactosidase. Under such conditions, lactose is hydrolyzed and the resulting galactose is polymerized via β-glycosidic bonds, yielding adducts containing 2–20 monomers [15]. In suckling pigs, [16, 17] GOS feeding was associated with altered intestinal microbiota composition, increased SCFA concentrations and tended to improve histomorphology. When fed to weanling pigs, GOS also increased fiber fermentation and SCFA concentrations accompanied by changes in microbial composition [18, 19].

Predicated on this previous research, the aim of the current experiment was to determine if supplementing prebiotic GOS to pigs both pre- and post-weaning would stimulate fermentation and facilitate weaning transition. Accordingly, our hypothesis was that GOS feeding would improve piglet growth performance by altering several parameters including intestinal morphology, SCFA concentrations, microbial composition and plasma cytokines.

## Materials and methods

### Animals and experimental design to assess pig growth performance

This experiment was conducted at the North Carolina State University Swine Educational Unit during May–July 2022. All animal procedures were approved by the Institutional Animal Care and Use Committee (protocol 23-142-01).

**Fig. 1:**
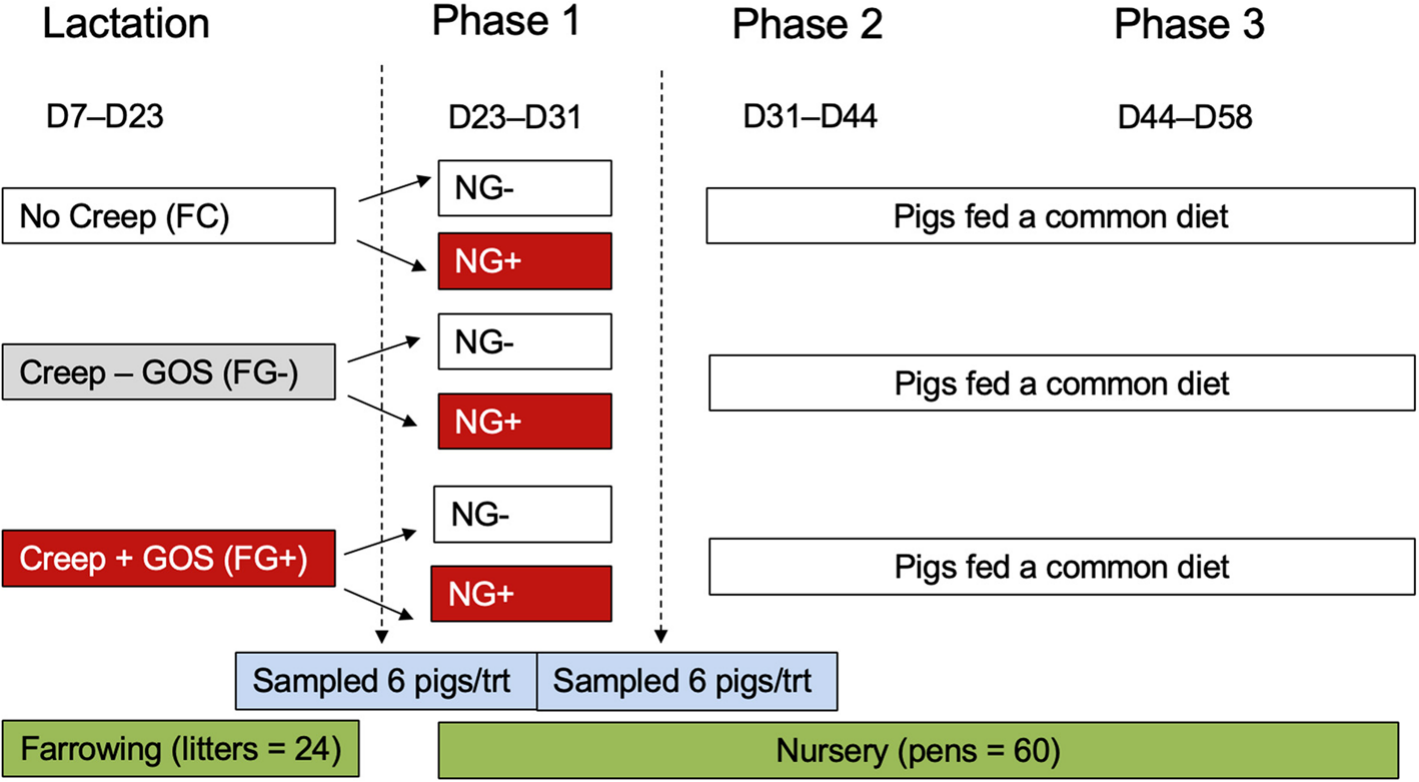
Experimental design illustrating peri-weaning feeding of GOS to piglets. Three dietary groups were fed during lactation followed by 2 dietary groups in the phase 1 nursery diet (3 × 2 factorial). Gruel creep diets fed during lactation were formulated without (FG–) or with 5% dietary GOS (FG+) and compared with no-creep controls (FC). Post-weaning phase 1 nursery diets were formulated without (NG–) or with 3.8% GOS (NG+). Pigs were sampled just prior to weaning (D22) or one-week post-weaning (D31) for intestinal measurements

The overall design of the experiment is illustrated in Fig. 1. Two creep diets were formulated (Table 1) to feed during the farrowing phase using highly digestible ingredients with the only difference being the addition of galactooligosaccharides at a 5.0% inclusion (GOS; designated FG+ diet), substituted for corn syrup solids (designated FG– diet). The GOS supplement was provided by an enriched whey permeate manufactured by Milk Specialties Global (Eden Prairie, MN, USA) containing 38% GOS. The 5% GOS inclusion rate was predicated on previous work [16] that supplemented 3.5% to formula-fed pigs. Because intake of supplemental creep was expected to be far less than exclusively formula-fed pigs we increased the inclusion rate accordingly.

**Table 1:** Creep diets supplemented to piglets during farrowing period containing no GOS (FG–) versus 5% supplemental GOS (FG+)^1^

Ingredient, %	FG–	FG+
Dried skim milk	27.00	27.00
Oat flour (ground)	20.00	20.00
Soy protein isolate	13.53	13.31
Whey permeate	21.44	21.44
Whey permeate (38% GOS)^2^	-	13.19
Corn syrup solids	13.41	-
Dextrose	2.25	2.25
Lecithin	0.43	0.43
Limestone	0.34	0.34
L-Lys HCl	0.35	0.35
L-Thr	0.30	0.30
DL-Met	0.18	0.18
L-Val	0.16	0.16
L-Trp	0.12	0.12
L-Ile	0.06	0.06
Vitamin premix^3^	0.19	0.19
Other^4^	0.47	0.47
Calculated composition		
DM, %	93.46	93.34
GE, kcal/kg	2,890	2,890
CP, %	25.50	25.00
Lactose, %	31.32	31.32
GOS, %	-	5.01
SID Lys, %	1.92	1.90
Ca, %	0.73	0.67
Total P, %	0.70	0.62

^1^Diets were formulated and manufactured by Milk Specialties Global (Eden Prairie, MN, USA)

^2^Spray dried GOS-containing whey permeate contained 38% GOS. Manufactured by Milk Specialties Global (Eden Prairie, MN, USA)

^3^Vitamin premix included per kg of diet: 24,900 IU of vitamin A as vitamin A acetate, 11,400 IU vitamin D_3_, 121 IU vitamin E, 0.014 mg vitamin B_12_, 5.21 mg riboflavin, 9.80 mg D-pantothenic acid as calcium pantothenate, 3.20 mg niacin, 0.10 mg biotin, and 1.11 mg pyridoxine

^4^Contained zeolex flow agent (0.25%), strawberry flavor (0.12%) and sucram (0.1%)

The diets were not formulated as complete feeds for suckling pigs, but as supplements to sow’s milk. Creep diets contained no antibiotics or growth promoting minerals and were fed ad libitum through novel gruel creep feeders as described by previous work [20].

Each diet was fed to eight litters and an additional eight control litters (designated FC) did not receive any creep feed (*n* = 24 litters total). Multiparous sows (> parity 2; Smithfield Premium Genetics; Yorkshire × Landrace × Duroc) were randomly assigned to treatment as they were placed into farrowing crates on D109 of gestation. Farrowing was induced with PGF2α injected on gestation D112. Cross-fostering was minimized but was used normalize litters to 12 pigs/litter by d 3 of age, resulting in a total of 288 piglets. On D7, litters began receiving one of the three farrowing treatments. These farrowing treatments continued until weaning at 23 days of age. Pig body weight, average daily gain and feed intake were measured at weekly intervals up until weaning. At weaning, pigs were blocked by weight and randomly allotted within farrowing treatment to two nursery treatments, yielding a 3 × 2 factorial treatment arrangement. Pigs were allotted to either a control phase 1 diet group lacking GOS (NG–) or to a GOS-supplemented phase 1 diet at an inclusion rate of 3.8% (NG+) and distributed among 60 pens with 3 pigs per pen. Diets were formulated to be as compositionally similar as possible and to meet the full nutritional requirements ([21]; Table 2) but lacked antibiotics or growth-promoting minerals. The 38% GOS-enriched whey permeate ingredient was substituted for normal whey permeate at 10% of the diet, pragmatically yielding the GOS supplementation rate of 3.8%. This substitution resulted in a slightly (3.8%) reduced ME content, but both diets exceeded NRC 2012 [21] energy recommendations. Furthermore, diets were rigidly formulated to supply equal carbohydrates (dextrin + lactose + GOS). Each diet was fed ad libitum to thirty pens for one-week (phase 1 period). Thereafter, all pigs received common phase 2 and phase 3 diets and post-weaning growth, including BW, ADG, ADFI and Gain:Feed, was recorded until D58. Fecal scores were recorded every day throughout the nursery phase using a 0 to 3 scale: (0) normal stool, (1) moist stool, (2) watery stool, (3) watery diarrhea as described in Goh et al. [22].

**Table 2:** Phase 1 diets fed during first week of nursery without (NG–) or with (NG+) supplementation of 3.8% GOS

Ingredient, %	NG–	NG+
Corn	19.18	19.6
Soybean meal (dehulled)	12.00	12.00
Cookie meal	10.00	10.00
Poultry meal	10.00	10.00
Whey permeate	24.00	14.00
Lactose	-	7.54
Whey permeate (38% GOS)^1^	-	10.00
Maltodextrin	7.96	-
Fish meal	4.00	4.00
Appetein^2^	4.00	4.00
Hamlet protein^3^	4.00	4.00
Poultry Fat	3.00	3.00
L-Lys^4^	0.56	0.56
DL-Met^5^	0.28	0.28
L-Thr^4^	0.20	0.20
L-Trp^4^	0.02	0.02
Limestone	0.40	0.40
Salt	0.22	0.22
Mineral premix^6^	0.15	0.15
Vitamin premix^7^	0.03	0.03
**Calculated composition, %**		
DM	91.71	92.48
ME, kcal/kg	3,545	3,409
CP	24.12	24.08
Lactose	19.20	19.20
GOS	0.00	3.80
Dextrin + lactose + GOS	27.31	27.31
SID Lys	1.50	1.50
Ca	0.88	0.86
STTD P	0.51	0.49

^1^Spray dried GOS-containing whey permeate contained 38% GOS. Manufactured by Milk Specialties Global (Eden Prairie, MN, USA)

^2^APC Proteins (Ankeny, IA, USA)

^3^Hamlet Protein Inc. (Findlay, OH, USA)

^4^CJ Bio (Fort Dodge, IA, UAS)

^5^Evonik (Essen, Germany)

^6^Mineral premix included per kg of diet: 33 mg Mn as manganous oxide, 100 mg Fe as ferrous sulfate, 110 mg Zn as zinc sulfate, 16.5 mg Cu as copper sulfate, 0.30 mg of I as ethylenediamine dihydroiodide, and 0.30 mg of Se as sodium selenite

^7^Vitamin premix included per kg of diet: 6,614 IU of vitamin A as vitamin A acetate, 992 IU vitamin D_3_, 19.8 IU vitamin E, 2.64 mg vitamin K as menadione sodium bisulfate, 0.03 mg vitamin B_12_, 4.63 mg riboflavin, 18.52 mg D-pantothenic acid as calcium pantothenate, 24.96 mg niacin, and 0.07 mg biotin

On D19 of farrowing, chromium oxide was added (3 g/kg) to the creep diets as a fecal marker. At one-day prior to weaning (D22 of age) and one-week post-weaning (D31 of age), 6 pigs per treatment (*n* = 54 total) were euthanized by AVMA-approved electrocution for measurement of intestinal parameters. Pigs of median weight were chosen, and pre-weaning pigs displaying green fecal swabs were chosen. Immediately prior to euthanasia blood was collected via jugular venipuncture (K2-EDTA Vacutainers) and centrifuged, and plasma was frozen at –80 °C for subsequent analysis.

Following exsanguination, a midline incision was made to remove the intestinal tract. A jejunal subsection, taken 60 cm from the pylorus and cut 5 cm in length, was fixed in 10% formalin for histology. Cecal digesta and cecal swabs (Sterile HydraFlock; Puritan Diagnostics) were collected and immediately frozen in liquid nitrogen and stored at –80 °C for subsequent SCFA and microbial analysis, respectively.

### Jejunal histology for morphology measures

Jejunal subsections were fixed in 10% formalin for 24 h, before a 70% ethanol wash and subsequent storage in 70% ethanol. Samples were further processed and epithelial morphometric measures including villus height, crypt depth, villus height:crypt depth, and villus surface area were obtained as described previously [20].

### Gas chromatography for cecal short chain fatty acid measurements

Frozen cecal digesta were thawed on ice, acidified with HCl and metaphosphoric acid, and short chain fatty acids were extracted and quantified using gas–liquid chromatography as previously described [23]. Specific SCFAs included acetate, propionate, butyrate, valerate, and branched-chain acids.

### Plasma cytokines

Luminex xMAP technology for multiplexed quantification of 13 porcine cytokines, chemokines, and growth factors were used. The multiplexing analysis was performed using the Luminex™ 200 system (Luminex, Austin, TX, USA) by Eve Technologies Corp. (Calgary, Alberta, Canada). Thirteen markers were simultaneously measured in the samples using the Porcine Cytokine 13-Plex Discovery Assay^®^ (MilliporeSigma, Burlington, Massachusetts, USA) according to the manufacturer’s protocol. Cytokines included IFN$\gamma$ IL-1$\alpha$, IL-1$\beta$, IL-Ra, IL-2, IL-4, IL-6, IL-8, IL-10, IL-12, IL-18, TNF$\alpha$, and GM-CSF.

### DNA extraction and 16S rRNA sequencing for downstream microbial analysis

DNA from cecal swabs was extracted using the DNeasy PowerSoil Pro kit (Qiagen, Hilden, Germany) following the manufacturer’s instructions. Cell lysis was performed on ice using a PowerLyzer 24 homogenizer at 2,000 r/min for 30 s, pausing for 30 s and homogenizing at the same speed for another 30 s. Concentrations of DNA were measured on a SpectraMax iD3 Microplate Reader (Molecular Devices, San Jose, CA, USA) and samples with A_260_/A_280_ of 1.8 were used. DNA samples were prepared for targeted sequencing with the Quick-16S Plus NGS Library Prep kit, specifically using primers for the V3–V4 region. Final PCR products were quantified with qPCR fluorescence readings and pooled based on equal molarity. The final pooled library was cleaned using a Select-a-Size DNA Clean & Concentrator, then quantified using a TapeStation (Agilent Technologies, Santa Clara, CA, USA) and QuBit (Thermo Fisher Scientific, Waltham, WA, USA). Sequencing was run on an Illumina MiSeq with a v3 reagent kit (600 cycles) with a 10% PhiX spike-in.

The 16S rRNA gene sequence analysis was conducted using R-Studio with the DADA2 pipeline. Low quality sequences were removed through the filtering and de-noising steps. Sequences were merged and chimeras were removed. An amplicon sequence variant (ASV) table was created, and taxonomic assignment was performed using the Silva reference database version 138.1. Microbial alpha diversity measures included Chao1, Shannon, and Simpson diversity. Beta diversity measures included principal coordinate analysis (PCoA) of center log ratio transformed data. A relative abundance bar graph at the Family level was constructed. Using the DESeq2 program, Log_2_FoldChanges in ASVs within the microbial community were analyzed and graphed when significant positive or negative changes were detected. For beta diversity measures, samples were trimmed to only include ASVs at 0.1% relative abundance and 20% prevalence. ASVs with less than four counts were discarded to remove potential sequencing errors. A center log ratio transformation was utilized for the PCoA plot using a Bray–Curtis distance matrix.

### Statistics

Statistical analysis of piglet growth performance, intestinal morphology and SCFA data were analyzed using the general linear model procedure in SAS (Cary, NC, USA), with litter or pen as the experimental unit. Data from the farrowing phase were analyzed according to a completely randomized design and data from the nursery phase were analyzed according to a randomized complete block design, with treatments arranged in 3 × 2 factorial design where farrowing treatment and nursery treatment were main effects. Effects of age (D22 vs. D31) and farrowing treatment also were modeled for cytokine data using a completely randomized design. Least square means were separated using a protected least significant difference test. Permanova tests in R-studio were used to determine whether microbial compositions were significantly different, while the lmer procedure was used to detect log fold ratio changes in specific microbiota. Significant differences were noted when *P* < 0.05 and trends identified when 0.05 < *P* ≤ 0.1.

## Results

### Pig growth performance

Pigs feed gruel creep feed during farrowing (FG– or FG+) were 6% heavier than controls (FC) on D14 and 12% heavier by D21 (Table 3; *P* < 0.05). Accordingly, ADG was 17% and 26% greater for creep-fed pigs than for controls during the first (D7–D14) and second (D14–D21) weeks of the trial.

**Table 3:** Effect of GOS supplementation within a gruel creep diet on piglet growth during in the farrowing phase^1^

Items	Treatment			SEM	*P*value
	FC	FG–	FG+		
Number of litters	8	8	8		
Number of pigs/litter	12	12	12		
Body weight, kg					
D7^2^	2.40	2.40	2.40	–	–
D14	3.84^a^	4.08^b^	4.08^b^	0.07	0.02
D21	5.52^a^	6.20^b^	6.19^b^	0.11	0.01
Average daily gain, g					
D7–14	205^a^	240^b^	240^b^	9.6	0.02
D14–21	240^a^	303^b^	301^b^	9.5	0.01
D7–21	223^a^	271^b^	270^b^	8.5	0.01
Average daily feed intake, g					
D7–14	–	13.0	9.7	1.0	0.06
D14–21	–	29.2	19.4	4.7	0.19
D7–21	–	21.1	14.5	2.7	0.13

^1^Litters fed gruel creep containing 5% GOS (FG+) were compared with controls given no creep (FC) and to litters feed creep without GOS (FG–)

^2^Data were analyzed with D7 body weight as a covariate. Unadjusted weights were 2.37, 2.62, and 2.23 for FC, FG–, and FG+ , respectively

^a,b^Least square means within a row lacking common superscript differ (*P* < 0.05)

In the nursery phase, there were no interactions detected between farrowing and nursery treatments; however, there were several main effects detected (Table 4; *P* > 0.1). By the end of the nursery phase (D58), pigs fed GOS pre-farrowing (FG+) were 1.61 kg heavier than FG– pigs and 1.85 kg heavier than FC pigs (*P* < 0.05). FG+ pigs tended to have a higher average daily gain during phase 3 and throughout the overall nursery phase (*P* = 0.09). There were no farrowing treatment effects detected in average daily feed intake or Gain:Feed (*P* > 0.1). When fed the phase 1 nursery diet containing GOS (NG+), pigs gained 34% faster (130 g/d) than NG– controls (97 g/d; *P* < 0.01). No effects of treatment were detected on ADG during phase 2 and phase 3 periods (*P* > 0.1), but there was an overall (D23–D58) increase in average daily gain with NG+ pigs growing 8% faster (*P* < 0.05). The increased growth rate was accompanied by increased feed intake, with NG+ pigs eating 11% more during phase 1 (*P* = 0.06), 8% more in phase 2 (*P* = 0.02), and 6% more overall (*P* = 0.06). Furthermore, a 15% increase in Gain:Feed was detected during phase 1, where NG+ had an efficiency of 0.69 compared to 0.60 for NG– pigs (*P* < 0.05). No differences in Gain:Feed were detected during any other phases (*P* > 0.1). There were no differences in fecal scoring (data not shown) detected throughout any of the phases of the nursery (overall average: 1.3 ± 0.5; *P* > 0.1).

**Table 4:** Effects of supplemental GOS in farrowing gruel creep feed and nursery phase 1 diet on growth performance in the nursery^1^

	Farrowing					Nursery			
Treatments	FC	FG–	FG+	SEM	*P* > F	NG–	NG+	SEM	*P* > F
Pens	20	20	20			30	30		
Body weight, kg									
D23	6.49^a^	7.16^b^	6.97^b^	0.06	0.01	6.88	6.81	0.06	0.33
D31	7.25^a^	8.13^b^	7.88^b^	0.11	0.01	7.66^x^	7.85^y^	0.07	0.06
D44	10.79^a^	11.39^ab^	11.68^b^	0.23	0.01	11.08^x^	11.50^y^	0.15	0.06
D58	17.76^a^	18.00^ab^	19.61^b^	0.48	0.02	18.03^x^	18.88^y^	0.32	0.06
Average daily gain, g/pig									
D23 to D31 (Phase 1)	105	122	114	12.5	0.59	97^a^	130^b^	8.3	0.01
D31 to D44 (Phase 2)	272	251	292	14.0	0.30	263	281	9.4	0.19
D44 to D58 (Phase 3)	498^x^	472^x^	566^y^	24.3	0.09	496	528	16.6	0.18
D23 to D58 (Nursery)	324^x^	310^x^	361^y^	13.0	0.09	318^a^	345^b^	8.7	0.04
Average daily feed intake, g/pig									
D23 to D31 (Phase 1)	159	191	162	10.4	0.10	161^x^	180^y^	6.9	0.06
D31 to D44 (Phase 2)	400	394	407	14.4	0.84	383^a^	417^b^	9.5	0.02
D44 to D58 (Phase 3)	718	758	801	33.3	0.15	746	772	22.1	0.43
D23 to D58 (Nursery)	426	448	457	14.5	0.18	430^x^	456^y^	9.6	0.06
ADG/ADFI, Gain:Feed									
D23 to D31 (Phase 1)	0.64	0.61	0.70	0.05	0.58	0.60^a^	0.69^b^	0.03	0.04
D31 to D44 (Phase 2)	0.68	0.65	0.71	0.02	0.34	0.68	0.67	0.02	0.67
D44 to D58 (Phase 3)	0.71	0.64	0.73	0.05	0.51	0.68	0.70	0.03	0.70
D23 to D58 (Nursery)	0.77	0.70	0.80	0.02	0.18	0.75	0.76	0.02	0.61

^1^Farrowing main effects compared control pigs fed no creep (FC) to pigs fed gruel creep without (FG–) or with 5% GOS (FG+). Nursery main effects compared pigs fed phase 1 diets without GOS (NG–) to those fed 3.8% GOS (NG+). Farrowing × Nursery interactions were not detected, *P* > 0.1

^a,b^Means within a row and treatment group lacking common superscript differ (*P* < 0.05)

^x,y^Means within a row and treatment group lacking common superscript tend to differ (*P* < 0.1)

### Jejunal morphology measures

In farrowing, there were no effects of treatment detected on villi length, crypt depth, villi:crypt ratio, or villus area (Table 5; *P* > 0.1). Post-weaning (Table 6), pigs fed GOS during phase 1 (NG+) tended to have increased surface area of villi compared to NG– pigs (*P* = 0.07). An interaction between farrowing and nursery treatments was detected with FG– pigs that received GOS in the nursery having increased villi length and villus area compared to FG– pigs that did not receive GOS in the nursery (Additional Fig. 1; *P* < 0.01).

**Table 5:** Jejunal morphology measured during farrowing (D22) in control pigs fed no creep (FC) compared with gruel-creep-fed pigs without (FG–) or with 5% supplemental GOS (FG+)^1^

Farrowing treatment	FC	FG–	FG+	SEM	*P* > F^2^
Villus height, μm	582	512	524	80.7	0.560
Crypt depth, μm	124	146	173	23.3	0.492
Villus:Crypt ratio	5.02	3.84	3.77	0.81	0.175
Villus area, μm^2^ × 10^–3^	221	216	195	207	0.680

^1^Data are means and SEM, *n* = 6

^2^Farrowing treatment main effects

**Table 6:** Jejunal morphology measured after weaning (D31) in pigs exposed to farrowing treatments (FC, FG–, FG+) followed by phase 1 nursery diets without (NG–) or with GOS (NG+)^1^

Farrowing treatment	FC		FG–		FG+		SEM	Ftrt^2^	Ntrt^3^	F × N^4^
Nursery treatment	NG–	NG+	NG–	NG+	NG–	NG+				
Villus height, μm	301^ab^	296^abc^	253^c^	343^a^	291^bc^	262^bc^	17.8	0.381	0.211	0.006
Crypt depth, μm	229	251	250	265	237	220	20.8	0.386	0.694	0.607
Villus:Crypt ratio	1.37	1.23	1.09	1.32	1.24	1.24	0.13	0.754	0.784	0.329
Villus area, μm^2^* ×* 10^–3^	103^b^	110^b^	92^b^	139^a^	106^b^	94^b^	9.3	0.258	0.070	0.011

^1^Data are means and SEM, *n* = 6

^2^Farrowing treatment main effect

^3^Nursery treatment main effect

^4^Farrowing × nursery treatment interaction

^a–c^Means within a row lacking a common superscript differ (*P* < 0.05)

### Cecal short chain fatty acid measurements

Prior to weaning, gruel creep feeding and supplemental GOS had no detectable effects on cecal SCFA composition measured on D22 (Table 7). However, measurements made on D31 (one-week post-weaning) showed a reduction in the molar proportion of acetate and an increase in the proportion of valerate when comparing FG– to FC pigs (Table 8; *P* < 0.05). Pigs fed GOS during farrowing (FG+) had the highest proportion of acetate and an intermediate level of valerate (*P* < 0.05). When GOS was fed in the phase 1 nursery diet, there was an increase in the molar proportion of butyrate and a corresponding decrease in the proportion of propionate in the cecum. (Table 8; *P* < 0.01). There were no differences detected in total SCFA concentrations (mmol/L) for either farrowing or nursery treatments (*P* > 0.1).

**Table 7:** Cecal short chain fatty acid composition measured during farrowing (D22) in control pigs fed no creep (FC) compared with gruel-creep-fed pigs without (FG–) or with 5% supplemental GOS (FG+)^1^

Farrowing treatment	FC	FG–	FG+	SEM	*P* > F
Items, mmol%					
Acetate	61.8	60.7	62.5	2.2	0.832
Propionate	22.3	25.0	22.6	1.5	0.389
Butyrate	7.48	6.97	7.74	0.8	0.777
Valerate	2.1	2.4	2.1	0.2	0.491
BCA^2^	6.4	4.9	4.3	0.7	0.118
Total, mmol/L	98.5	87.9	87.7	12.7	0.791

^1^Data are means and SEM, *n* = 6

^2^ Branched-chain acids: isobutyrate plus isovalerate

**Table 8:** Cecal short chain fatty acid composition measured after weaning (D31) in pigs exposed to farrowing treatments (FC, FG–, FG+) followed by phase 1 nursery diets without (NG–) or with GOS (NG+)^1^

Treatment	Farrowing					Nursery			
	FC	FG–	FG+	SEM	*P* > F	NG–	NG+	SEM	*P* > F
Items, mmol%									
Acetate	60.3^a^	55.1^b^	61.3^a^	1.5	0.014	57.5	60.3	1.2	0.105
Propionate	28.0	28.2	25.8	1.1	0.243	30.1^a^	24.5^b^	0.9	0.001
Butyrate	10.4	13.4	11.5	1.0	0.128	10.1^a^	13.4^b^	0.8	0.011
Valerate	0.8^a^	2.5^b^	1.4^b^	0.3	0.007	1.5	1.5	0.3	0.690
BCA^2^	0.5	0.8	0.2	0.2	0.129	0.8	0.1	0.1	0.010
Total, mmol/L	102.9	98.5	108.5	11.2	0.822	104.8	101.8	9.1	0.823

^1^Data are means and SEM; *n* = 18 per farrowing treatment and *n* = 36 per nursery treatment. No farrowing × nursery interactions were detected (*P* > 0.1) so only main effects are shown

^2^Branched chain acids: isobutyrate plus isovalerate

^a,b^Means within a row lacking a common superscript differ (*P* < 0.05)

### Plasma cytokines

**Fig. 2:**
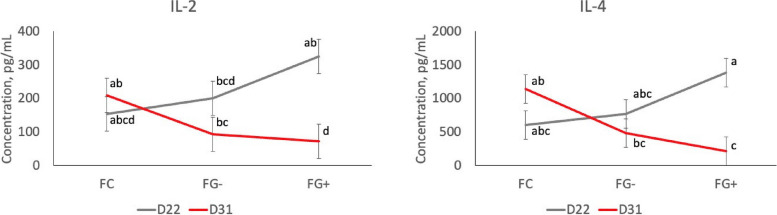
Plasma IL-2 and IL-4 concentrations in control pigs (FC) versus pigs fed gruel creep without (FG–) or with GOS (FG+), measured pre- (D22) and post-weaning (D31). Farrowing treatment × age interaction, *P* < 0.05. ^a–d^ Means lacking a common superscript differ, *P* < 0.05. Similar interactions were observed for IL-Ra and IL-18 (Additional Table 1)

**Fig. 3:**
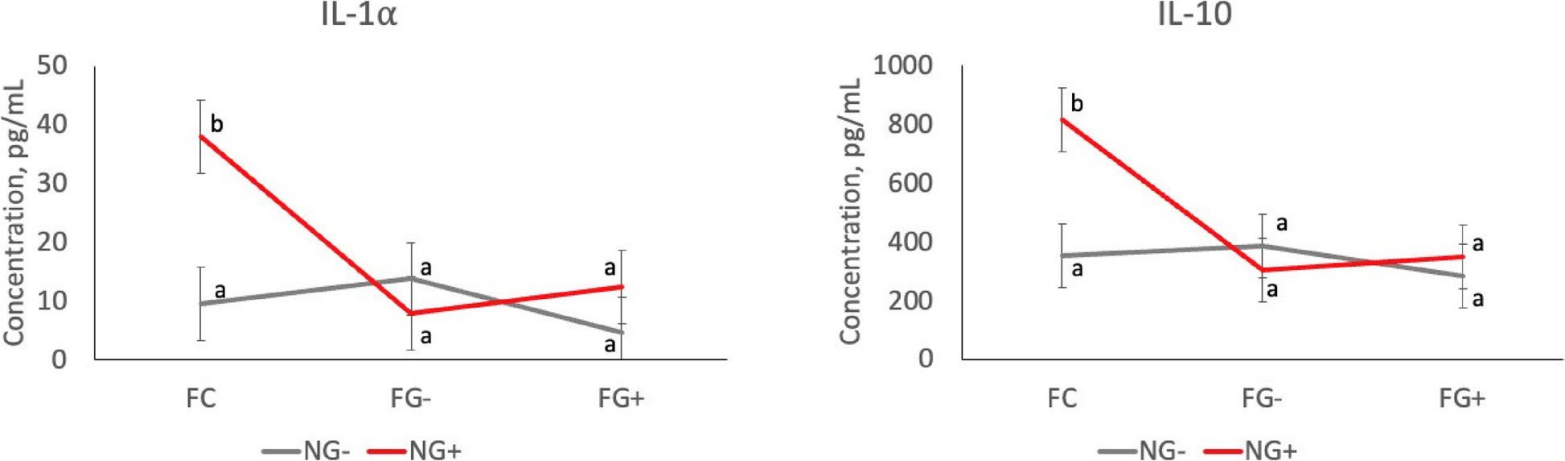
Plasma IL-1α and IL-10 concentrations in pigs exposed to farrowing treatments (FC, FG–, FG+) followed nursery diets without (NG–) or with GOS (NG+), measured one-week post-weaning (D31). Farrowing × nursery treatment interaction, *P* < 0.05. ^a,b^Means lacking a common superscript differ (*P* < 0.05). Similar interactions were observed for IL-2, IL-4, and IL-18 (Additional Table 2)

Irrespective of nursery treatment, several plasma cytokine concentrations (IL-2, IL-4, IL-Ra, and IL-18) displayed differential responses to farrowing treatment with piglet age (Fig. 2 and Additional Table 1). Specifically, pigs fed creep with GOS (FG+) had highest concentrations pre-weaning (D22) but lowest concentrations post-weaning (D31; farrowing treatment × age interaction, *P* < 0.05). Several cytokines (IL-1α, IL-2, IL-4, IL-10, and IL-18) measured post-weaning (D31) also showed interdependence of farrowing and nursery diet effects (Fig. 3 and Additional Table 2). Specifically, pigs supplemented with GOS in the nursery diet (NG+) showed elevated concentrations, but only for pigs that did not receive creep feed during farrowing (FC; farrowing × nursery treatment interaction, *P* < 0.05).

### Microbial analysis: alpha diversity, beta diversity, taxonomic analysis, microbial abundance

**Fig. 4:**
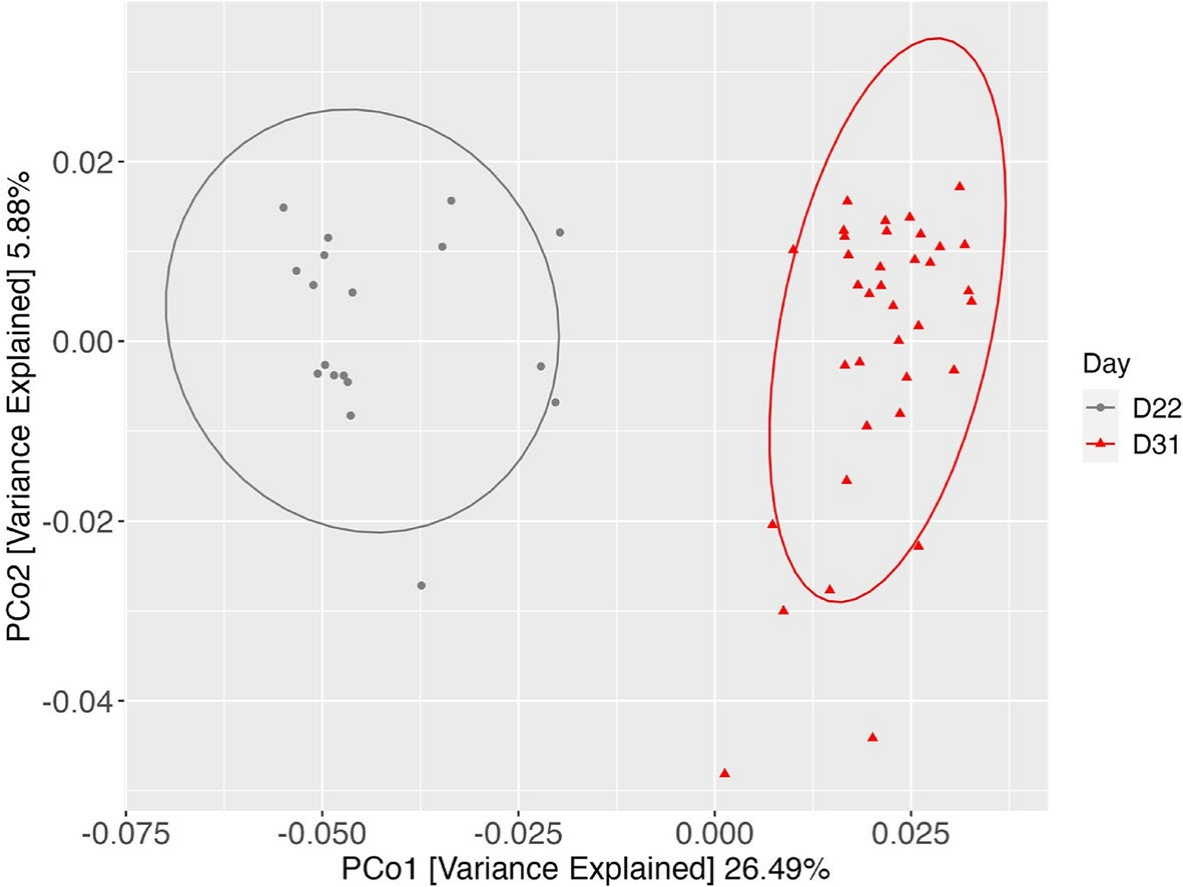
Beta diversity of cecal microbial communities in pre- (D22) vs. post-weaned (D31) pigs. Principal coordinate analysis (PCoA) was used based on Bray–Curtis dissimilarity matrix, *P* < 0.05

Sequencing of the 16S rRNA genes in the samples produced a total of 7,363,701 reads after filtering and removal of chimeric sequences with an average of 136,364 ± 16,612 reads. Samples were subset by day, and main effects of pre-weaning and post-weaning diets were assessed. Cecal microbiota clustered strongly when contrasting pre- and post-weaning samples based on principal coordinate analysis, with no overlap observed (Fig. 4, *P* < 0.01).

**Fig. 5:**
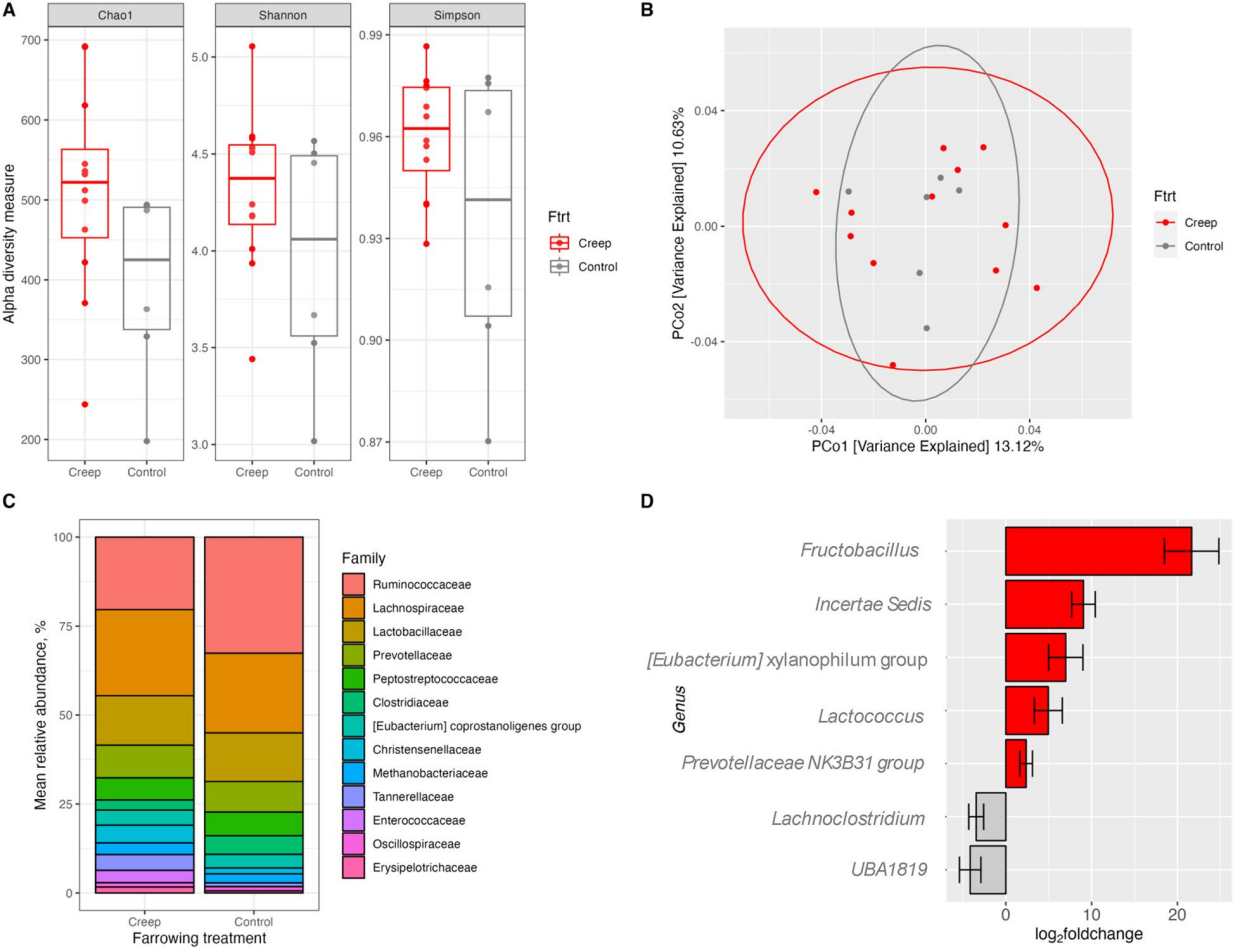
Microbial analysis in the pre-weaning (D22) cecum of gruel creep vs. control fed pigs. Creep includes pigs fed gruel creep feed both without (FG–) or with GOS (FG+) while control pigs were not fed creep feed. **A** Alpha diversity measures of Chao1 (*P* < 0.05), Shannon (*P* > 0.1), and Simpson (*P* = 0.09) metrics. **B** Beta diversity based on Bray–Curtis dissimilarity matrix, *P* > 0.1. **C** Family-level taxonomy distributions. **D** Log_2_fold changes in microbial genera of creep-fed vs. control pigs, *P* < 0.05

In the pre-weaning (D22) cecum (Fig. 5), further analyses were performed comparing only creep-fed pigs to control pig because there were no effects detected between FG– and FG+ treatments. Within sample alpha diversity assessment showed an effect on Chao1 diversity (*P* < 0.0), being greater in creep-fed pigs, but no effects for Shannon and Simpson diversity were detected (Fig. 5A; *P* > 0.1). There was no difference detected in β-diversity (Fig. 5B; *P* > 0.1). Descriptive taxonomic analysis at the family level (Fig. 5C) had no significant shifts (*P* > 0.1).

Ruminococcaceae, Lachnospiraceae, and Lactobacillaceae were the predominant three families across both treatments. At the genus level, when comparing creep fed pigs to control pigs, there was an increase in relative abundance of *Fructobacillus*, *Incertae Sedis* [Ruminococcaceae], [*Eubacterium*] *xylanophilum* group, *Lactococcus*, and *Prevotellaceae NK3B31* group and a decrease in *Lachnoclostridium* and *UBA1819* [Ruminococcaceae] (Fig. 5D; *P* < 0.05).

**Fig. 6:**
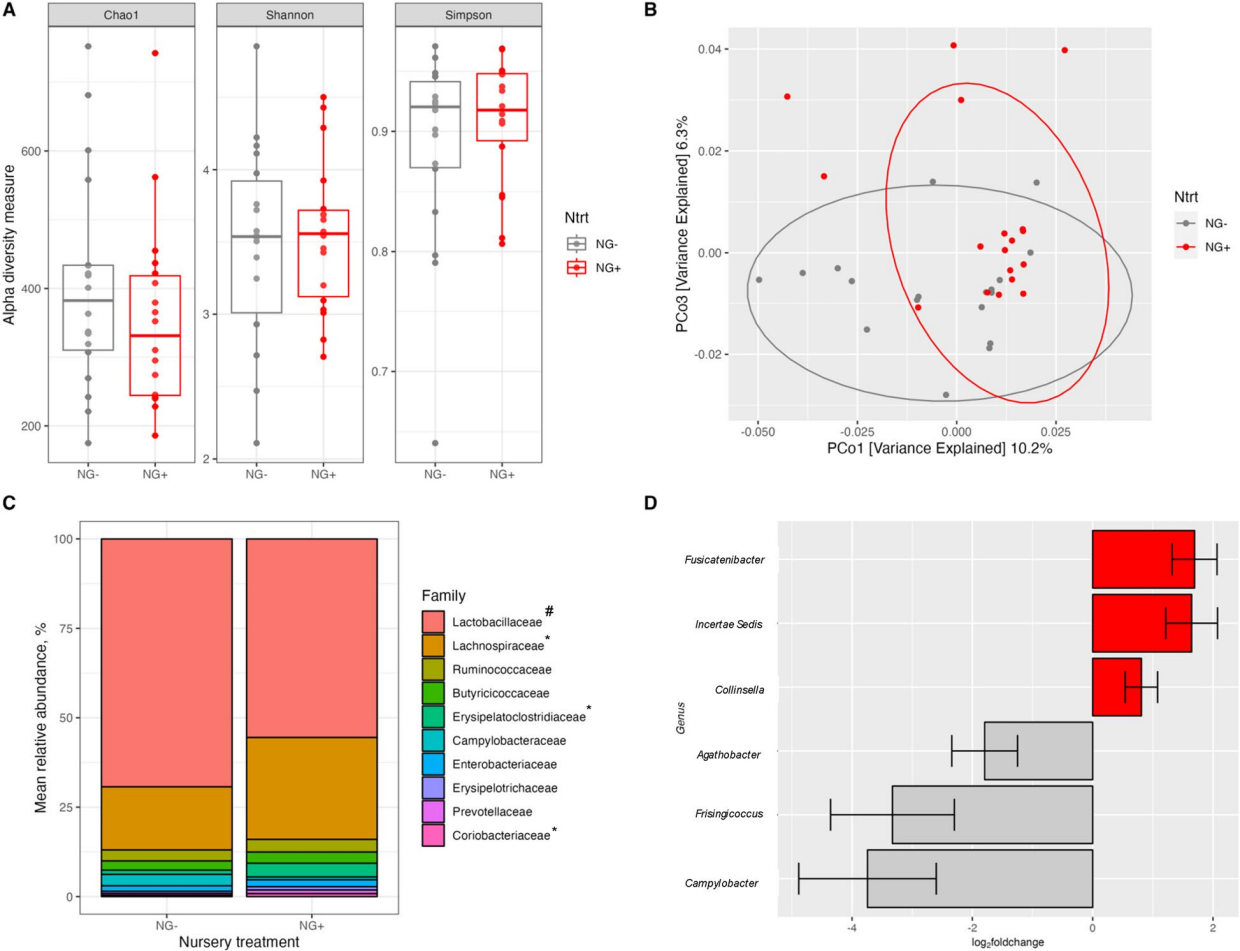
Microbial analysis in the post-weaning (D31) cecum of pigs fed a phase 1 nursery diet without (NG–) or with supplemental GOS (NG+). **A** Alpha diversity measures of Chao1 (*P* > 0.1), Shannon (*P* > 0.1), and Simpson (*P* > 0.1) metrics. **B** Beta diversity based on Bray–Curtis dissimilarity matrix, *P* < 0.05.** C** Family-level taxonomy distributions. (^#^NG– > NG+ , ^*^NG+ > NG–) **D** Log_2_fold changes in microbial genera of NG+ vs. NG– pigs, *P* < 0.05

In the post-weaning (D31) cecum (Fig. 6), analyses compared GOS fed pigs (NG+) to controls (NG–). There were no GOS effects detected for Chao1, Shannon or Simpson alpha-diversity metrics (Fig. 6A; *P* > 0.1), but beta diversity PCoA revealed a significant shift of microbial communities due to GOS supplementation (Fig. 6B, *P* < 0.01). Descriptive taxonomic analysis at the family level (Fig. 6C), showed that GOS supplementation (NG+) increased proportions of Lachnospiraceae, Erysipelatoclostridiaceae and Coriobacteriaceae by 11%, 2.7% and 0.5%, respectively, whereas Lactobacillaceae proportion increased in controls (NG–) by 14% (*P* < 0.05). Furthermore, there were several shifts in bacterial relative abundance at the genus level (Fig. 6D, *P* < 0.05). Specifically, dietary GOS increased cecal *Fusicatenibacter*, *Incertae Sedis* [Ruminococcaceae], and *Collinsella*, but decreased *Agathobacter*, *Frisingicoccus*, and *Campylobacter.*

## Discussion

Creep feeding can increase piglet weaning weight, leading to improved nursery growth performance. Our aim in this study was to examine whether a prebiotic (GOS) supplement into a gruel creep diet fed pre-weaning could have a synergistic effect when subsequently included in a phase 1 nursery diet fed post-weaning.

The use of supplemental liquid milk replacer during lactation is not novel [6, 24]; whereas gruel creep feeding is less studied. However, there are some studies that report increased feed intake as well as improved weaning weight when supplemental liquid gruel creep is provided [20, 25, 26]. A study by Heo et al. [27] reported average daily gain ranging from 196 g/d to 221 g/d between D14–D21 in farrowing. Comparatively, we observed higher ADG during the same period of 302 g/d. Sustained effects of creep feeding have also been debated, for example, Muns and Magowan [28] reported an increase in weaning weight and increased ADFI in the beginning of nursery, but creep effects over time were dissipated due to increased variability between treatments. In contrast, we observed a progressive increase in body weight between FG+ and FC pigs, whereas FG– fed pigs became converged with the FC group over time. It is remarkable that GOS supplementation in farrowing produced a delayed and substantial effect on growth performance in the later phases of the nursery.

There is a plethora of information on the benefits of prebiotics in nursery feeds, but limited data with prebiotic supplementation to piglets during farrowing. Usually, prebiotics are gavaged as described in Tian et al. [29] or added in milk replacer for young piglets [16, 17]. While Tian et al. [29] and Berding et al. [16] observed improved growth, Eudy et al. [17] saw no difference between treatment groups. The latter is in line with our findings where GOS had no effect on feed intake or ADG in farrowing, but instead, the supplementation of gruel creep feeding drove greater pig performance. Nursery prebiotic supplementation studies usually focus on shifts in microbial diversity and SCFA profiles, but there may also be improvements in growth performance. Both San Andres et al. [30] and Li et al. [31] reported that prebiotic supplementation to post-weaned pigs did not alter ADG, but ADFI and G:F ratios were improved. We observed improvements of ADG, ADFI and G:F with GOS supplementation. It is particularly intriguing to consider how the substitution of a non-digestible prebiotic oligosaccharide (GOS) for an easily digestible substrate (maltodextrin) improved feed efficiency. We suggest that alteration of gut microbial communities, fermentation and intestinal health may contribute.

The inclusion rate of prebiotic substances can vary based on the ingredient added. Some studies have supplemented selected bioactive prebiotics in as small of an amount of 0.1% [8, 22], while in this study GOS supplementation was much higher at 5.0% and 3.8% of gruel creep and nursery diets, respectively. These higher feeding rates were based on the intention to provide a sufficient amount of substrate to fuel fermentation and were predicated on piglet formula-feeding experiments with inclusion rates up to 3.5% [16, 17].

Villus length and crypt depth are widely used as markers of gut health. Weaning stress leads to villus atrophy in the intestine while crypt depths lengthen by cause of increased proliferation of cells. Van Beers-Schreurs et al. [32] found that villus atrophy is specifically attributed to the decrease in feed intake immediately post-weaning, where piglets shift from a highly digestible milk based diet to a fibrous, less digestible nursery feed. In this study we hypothesized that supplemental creep feed during farrowing would increase piglet feed intake in the nursery, but this was not supported by the data. There were no differences detected in ADFI between any of the farrowing treatments in the first week of the nursery. Jointly, there also were no differences detected in villus height or crypt depth at D31. However, there was an interaction between farrowing and nursery treatments where FG– pigs that received GOS in the nursery had longer villi than FG– pigs receiving no GOS.

SCFAs are directly affected by the addition of fermentable prebiotics which fuel microbial growth in the hindgut. Because mammalian enzymes cannot degrade the fibrous plant material, only bacterial digestion can break down beta-glycosidic bonds present in GOS. Tzortzis et al. [18] found that the addition of GOS at both 1.6% and 4% inclusions increased amounts of lactic and acetic acid in the proximal colon, while in the distal colon, butyric acid increased. They also found a reduced pH in the proximal colon, suggesting that the oligosaccharide remained intact until it was fermented in the hindgut. In another study testing dietary fiber inclusion in creep feed [33], long-chain arabinoxylans increased propionate in the cecum. Pre-weaning, there were no effects detected in the SCFA profile, although this may be due to the comparatively low intake of gruel creep versus the predominant consumption of sow’s milk. In the nursery, we observe main effects on propionate and butyrate proportions decreasing and increasing for NG+ pigs, respectively. This difference may be due to increased fiber intake post-weaning, where the only sustenance provided is the nursery diet. An in vivo study of soy galactooligosaccharide (raffinose + stachyose) and trans-galactooligosaccharide (TOS) additions and their effect on ileal SCFA found that the soybean oligosaccharides produced a significant increase in propionate and butyrate, whereas TOS had no changes detected [34]. In contrast, Li et al. [8] showed that mannan-oligosaccharide supplementation decreased both butyrate and propionate concentrations, while increasing acetate. It is important to note that different oligosaccharides may affect microbial populations differently, thereby affecting SCFA profiles in contrasting ways. Furthermore, in vitro fermentation studies of GOS are necessary to verify changes in SCFA production, rather than concentration.

Of the SCFAs, butyrate is of prime interest due to many studies citing positive effects of protected butyrate supplementation to pigs. Protected butyrate supplementation has been observed to increase villus area, therefore increasing nutrient absorption, as well as to increase sucrase activity in the ileum [35]. Butyrate supplementation improved growth performance, improved the intestinal microflora, and most importantly improved intestinal barrier function [36, 37]. While not directly supplementing butyrate, GOS feeding increased the molar proportion of butyrate in our study and was positively correlated with improved ADG. The increase in relative butyrate concentration may have had several impacts on the mucosal lining, tight junction function, or intestinal permeability. Further experimentation is required and again, in vitro experimentation would be needed to assess the shift in butyrate to verify that production has increased.

Immunological analysis revealed a trend in several pro- and anti-inflammatory cytokines in the peri-weaning period. In general, pre-weaned pigs in the FC group had the lowest cytokine levels, then FG–, followed by FG+ . However, post-weaning, the reverse was observed where FG+ had the lowest cytokine concentrations compared with FC pigs. This was expected, because the addition of a gruel creep diet introduced non-milk, less digestible ingredients like oats and soy isolate, which can elicit an increased immune response [38, 39]. There is debate whether an increased or decreased immune response is positively associated with growth, but less energy expenditure to the immune system may increase energy partitioned for growth in young pigs.

Post-weaning, the addition of GOS displayed an interaction between farrowing and nursery treatments. While FG– and FG+ pigs had no discernable difference in cytokine levels post-weaning, the FC group was greatly influenced by nursery treatment; NG+ pigs had markedly higher levels of both pro- and anti-inflammatory cytokines compared to the NG– pigs. This influence is a typical observation of oligosaccharide inclusion in the diet post-weaning. Soy galactooligosaccharides such as raffinose and stachyose are known to decrease digestibility as well as have a negative influence on gut immunity [40]. Galactooligosaccharides derived from lactose, like GOS in our study, may have a similar effect when introduced to pigs that have not been exposed to any prior fiber or GOS.

The microbiome of the hindgut is affected by the addition of prebiotics, however it is imperative to emphasize that the biggest contributor to microbial changes in piglets is the weaning event. Shifts from highly digestible milk nutrients to a complex fibrous diet is the driving force for rapid maturation of the young piglet microbiome. Both Bian et al. [41] and Gueverra et al. [42] agree that weaning significantly effects the microbial diversity of piglets, while the former notes it is more important than breed or nursing sow. Our PCoA analysis, clustering bacterial communities of pre-weaned vs. post-weaned pigs, is very similar to the findings in Guevarra et al., [42] where there is little if any overlap between treatment groups. Because our data agreed with previous work, we further examined if prebiotic GOS supplementation would have an effect in the pre- or post-weaned pigs.

Alpha diversity, or richness, is a measure of mean species diversity within a sample, therefore the higher number of unique ASVs, the greater the richness. Supplementation of prebiotics would theoretically increase richness due to an increase in fermentable substrate for microbiota that would otherwise be deprived. Guevarra et al. [43] noted that alpha diversity indices increase over time while the variability of microbiota among individual piglets decreases. We found that there were no differences in alpha diversity between FG– and FG+ pigs, but when combining gruel creep treatments, there was a significant increase in ASVs. These results were expected because the addition of a fibrous creep introduced fermentable substrate. Post-weaning, there were no effects detected of treatment on alpha diversity, which parallels a study by van Hees et al. [33], where none of their fiber supplemented treatments had any detectable difference. However, several studies find that more time may be needed for gut microbial changes to reflect the increase fiber in the diet [44, 45]. In the future, samples taken over time throughout the nursery may reveal a trend in the change of richness in the hindgut.

Pre-weaning PCoA analysis did not reveal any overall shifts in microbial communities. This was not surprising because most of the nutrients pigs received were from sow’s milk. Gruel creep consumption was comparatively low, and while there was fiber included in this diet, it is possible that insufficient amounts were consumed to elicit detectable shifts in the microbial community; however, there were alterations in several genera (discussed below). Beta diversity measured post-weaning revealed a significant shift in the community (*P* < 0.05), which may be attributed to the increased intake of GOS. In a study observing the effects of *Bacillus subtilus* or antibiotics on the piglet microbiome, they found that sampling site was the biggest influencer of shifts in beta diversity, but probiotic and antibiotic supplementation also shifted microbial communities [46]. This is comparable to the results we observed, where NG+ pigs differentiated from NG– pigs. In contrast, another study utilizing phytobiotic supplementation to weaned pigs failed to detect a difference between treatment and control groups [47]. PCoA analysis can be deceptive because the type of dissimilarity matrix used may affect the outcomes. More research is needed to verify the hypothesis that bacterial communities continue to change over time based on a phase 1 inclusion of GOS. It would also be of interest to investigate whether GOS supplementation in subsequent diet phases would further alter the gut microbiome and benefit gut health.

Addition of GOS in the pre-weaning gruel diets did not significantly alter the relative abundance of microbiota between FG– and FG+ pigs. However, when the two treatments were combined and compared to controls (FC), there were several log_2_fold changes in specific microbiota. *Fructobacillus*, of the family Lactobacillaceae, had the largest log_2_fold change of 21.6, followed by *Incertae Sedis*, family Ruminococcaceae with 8.9, and then *Eubacterium xylanophilum* group of family Lachnospiraceae at 6.9. All three are common families that increase in abundance post-weaning. Due to the nature of the gruel creep, there were several fermentable ingredients including oats, soy isolate, and GOS. When compared to FC pigs, it is reasonable that the creep diet would support increases in microbes that utilize fiber as a substrate and increase in the nursery. Frese et al. [9] found that due to the shift in diet, Lactobacillaceae, Ruminococcaceae, and Lachnospiraceae increased in relative abundance when comparing pre- vs. post-weaned bacterial populations in feces. This aligns with findings we observed where three families increased because of the introduction to a new diet in the form of gruel creep supplementation. The two microbes *Lachnoclostridium* and *UBA1819*, while also from the same families decreased in gruel fed pigs, but the magnitude of change was lower than the change observed in organisms that increased. Log_2 _fold changes of post-weaned pigs revealed several increases in *Fusicatenibacter*, *Incertae sedis* and *Collinsella* – all of which are common hindgut fermenters in healthy pigs. Isolated *F. saccharivorans* produces enzymes that break down sugars, including α- and β-galactosidases [48], with end metabolites such as acetate, formate, lactate, and succinic acid. *Collinsella aerofaciens*, of the phylum Actinobacteriota, is a butyrate producer that has been correlated with increased propionate and butyrate production in vivo [49]. A similar amount of microbiota decreased relative abundance in NG+ pigs compared to NG–, with shifts of comparable magnitudes, such as *A. rectalis* from the family Lachnospiraceae. *Agathobacter,* which are Gram positive microbes, mainly produce butyrate, acetate, hydrogen, and lactate [50]. An identical trend for *F. caecimuris* and *Campylobacter* was found where the former, Lachnospiraceae organism, was generally thought to be a beneficial gut fermenter, producing acetate and butyrate [51]. *Campylobacter*, which decreased the most in our treatment group, is a Gram-negative pathogenic bacteria that is correlated with diarrhea, irritable bowel syndrome, and enterocolitis. Primarily focused on the poultry world, there is concern over *Campylobacter* to have resistance to several antimicrobials like quinolones or tetracyclines [52].

The importance of individual microbiota must not be understated, but the microbiome as a multivariate “system” is the most adept interpretation of the gut microbial ecosystem. Addition of feed additives such as GOS may shift several genera in the hindgut, but microbiota do not exist in isolation and the network of microbes together may inform phenotypic changes [53, 54]. As sequencing technologies advance in the form of shotgun sequencing or the adaptation of other “omics” approaches, the dynamics of microbe-host interactions can be viewed with greater clarity.

## Conclusion

Pre-weaning improvements in piglet performance, intestinal health, and altered microbiota were associated with gruel creep feeding, irrespective of GOS supplementation. However, GOS had a significant impact post-weaning, increasing feed intake, growth and efficiency as well as shifting the microbiota, resulting in a higher proportion of butyrate. Overall, the bacteria that increased were beneficial hindgut fermenters including *Fructobacillus* and *Fusicatenibacter*, whereas other bacteria such as *Campylobacter* decreased. Further research is recommended on the interplay of microbes with changes in phenotypic expression and how they affect swine growth and gut health. Future directions for prebiotic use may include a longer inclusion period or titrations of GOS, based on results of the present study.

### Supplementary Information

**Additional file 1: Additional Table 1.** Plasma cytokine concentrations in control pigs (FC) versus pigs fed gruel creep without (FG–) or with GOS (FG+), measured pre- (D22) and post-weaning (D31).

**Additional file 2: Additional Table 2.** Plasma cytokine concentrations in pigs exposed to farrowing treatments (FC, FG–, FG+) followed nursery diets without (NG–) or with GOS (NG+), measured one-week post-weaning (D31).

** Additional file 3: Additional Fig. 1.** Jejunal morphology^1^ after weaning (D31) in pigs exposed to farrowing treatments (FC, FG–, FG+) followed by phase 1 nursery diets without (NG–) or with GOS( NG+).

## Data Availability

Data available at Dryad: 10.5061/dryad.0k6djhb5w. Microbiota sequence data available at NCBI. Submission ID: SUB14387485; BioProject ID: PRJNA1103211.

## Abbreviations

ADG: Average daily gain
ADFI: Average daily feed intake
ASV: Amplicon sequence variant
BCA: Branched chain acids
BW: Body weight
FC: Farrowing control
FG–: Farrowing creep without GOS
FG+: Farrowing creep with GOS
G:F: Gain:feed
GM-CSF: Granulocyte–macrophage colony-stimulating factor
GOS: Galactooligosaccharide
IFNγ: Interferon gamma
IL: Interleukin
NG–: Nursery diet without GOS
NG+: Nursery diet with GOS
PCoA: Principal coordinate of analysis
PGF2α: Prostaglandin F2 alpha
SCFA: Short chain fatty acid
SEM: Standard error mean
TNFα: Tumor necrosis factor alpha
