# Supplementary material for: Prebiotic galactooligosaccharide improves piglet growth performance and intestinal health associated with alterations of the hindgut microbiota during the peri-weaning period
Source: J Anim Sci Biotechnol. 2024 Jun 13;15:88. doi: 10.1186/s40104-024-01047-y (PMC11170840; doi:10.1186/s40104-024-01047-y)
Supplement: Supplementary file 1 — Additional file 1: Additional Table 1. Plasma cytokine concentrations in control pigs (FC) versus pigs fed gruel creep without (FG–) or with GOS (FG+), measured pre- (D22) and post-weaning (D31). [file 40104_2024_1047_MOESM1_ESM.docx]

Additional Table 1. Plasma cytokine concentrations in control pigs (FC) versus pigs fed gruel creep without (FG-) or with GOS (FG+), measured pre- (D22) and post-weaning (D31).

|  | Treatment | | | | | |  |  |  | *P* > F | | |
| --- | --- | --- | --- | --- | --- | --- | --- | --- | --- | --- | --- | --- |
|  | FC | | FG- | | FG+ | |  | SEM |  | Trt^1^ | Age | T x A^2^ |
| Items, pg/mL | D22 | D31 | D22 | D31 | D22 | D31 |  |  |  |  |  |  |
| IFNγ | 4,883 | 4,018 | 5,503 | 4,432 | 7,061 | 4,022 |  | 557 |  | 0.177 | 0.001 | 0.140 |
| IL-1α | 24.6 | 23.7 | 25.6 | 10.9 | 38.2 | 8.4 |  | 7.8 |  | 0.741 | 0.027 | 0.204 |
| IL-1β | 193.0 | 296.5 | 226.6 | 184.7 | 431.1 | 146.6 |  | 75.4 |  | 0.578 | 0.242 | 0.046 |
| IL-1ra | 820^a^ | 1,690^b^ | 1,422^b^ | 782^a^ | 626^a^ | 618^a^ |  | 225 |  | 0.023 | 0.695 | 0.007 |
| IL-2 | 152.8^bcd^ | 208.1^ab^ | 200.8^bcd^ | 93.2^bc^ | 325.7^ab^ | 72.3^d^ |  | 58.2 |  | 0.688 | 0.041 | 0.040 |
| IL-4 | 598^abc^ | 1,136^ab^ | 763^abc^ | 477^bc^ | 1,385^a^ | 208^c^ |  | 286 |  | 0.686 | 0.203 | 0.018 |
| IL-6 | 119.3 | 122.3 | 121.9 | 76.4 | 171.7 | 69.6 |  | 21.6 |  | 0.541 | 0.011 | 0.067 |
| IL-8 | 52.8 | 78.9 | 48.2 | 53.6 | 91.3 | 56.5 |  | 12.5 |  | 0.211 | 0.916 | 0.062 |
| IL-10 | 567 | 583 | 599 | 344 | 1,010 | 315 |  | 158 |  | 0.511 | 0.022 | 0.093 |
| IL-12 | 799 | 1,162 | 934 | 975 | 767 | 1,191 |  | 150 |  | 0.983 | 0.033 | 0.425 |
| IL-18 | 1,413 | 1,960 | 1,802 | 1,254 | 2,967 | 981 |  | 353 |  | 0.475 | 0.029 | 0.004 |
| TNFα | 225.2^a^ | 168.1^abc^ | 187.0^abc^ | 120.7^bcd^ | 183.3^abc^ | 92.4^cd^ |  | 28.5 |  | 0.118 | 0.004 | 0.837 |
| GM-CSF | 91.0 | 92.8 | 80.4 | 71.7 | 94.9 | 75.6 |  | 8.8 |  | 0.194 | 0.242 | 0.513 |

^1^ Treatment.

^2^ Treatment by age interaction.

^abcd^ Means within a row lacking a common superscript differ (*P* < 0.05).
