# Supplementary material for: Prebiotic galactooligosaccharide improves piglet growth performance and intestinal health associated with alterations of the hindgut microbiota during the peri-weaning period
Source: J Anim Sci Biotechnol. 2024 Jun 13;15:88. doi: 10.1186/s40104-024-01047-y (PMC11170840; doi:10.1186/s40104-024-01047-y)
Supplement: Supplementary file 2 — Additional file 2: Additional Table 2. Plasma cytokine concentrations in pigs exposed to farrowing treatments (FC, FG–, FG+) followed nursery diets without (NG–) or with GOS (NG+), measured one-week post-weaning (D31). [file 40104_2024_1047_MOESM2_ESM.docx]

Additional Table 2. Plasma cytokine concentrations in pigs exposed to farrowing treatments (FC, FG-, FG+) followed nursery diets without (NG-) or with GOS (NG+), measured one-week post-weaning (D31).

| Farrowing Treatment: |  | FC | | FG- | | FG+ | |  | SEM | Ftrt^1^ | Ntrt^2^ | F × N^3^ |
| --- | --- | --- | --- | --- | --- | --- | --- | --- | --- | --- | --- | --- |
| Nursery Treatment: |  | NG- | NG+ | NG- | NG+ | NG- | NG+ |  |  |  |  |  |
| Items, pg/mL |  |  |  |  |  |  |  |  |  |  |  |  |
| IFNγ |  | 4,167 | 3,870 | 4,419 | 4,446 | 4,253 | 3,793 |  | 343 | 0.395 | 0.393 | 0.773 |
| IL -1α |  | 9.45^a^ | 37.9^b^ | 13.7^a^ | 7.9^a^ | 4.5^a^ | 12.3^a^ |  | 6.2 | 0.045 | 0.058 | 0.032 |
| IL-1 β |  | 166 | 427 | 215 | 155 | 139 | 152 |  | 73.4 | 0.119 | 0.244 | 0.088 |
| IL -1ra |  | 1,709 | 1,671 | 851 | 713 | 546 | 692 |  | 278 | 0.001 | 0.965 | 0.876 |
| IL-2 |  | 91.8^a^ | 324.3^b^ | 111.6^a^ | 74.7^a^ | 45.5^a^ | 99.1^a^ |  | 51.2 | 0.027 | 0.056 | 0.040 |
| IL-4 |  | 378^a^ | 1,894^b^ | 454^a^ | 500^a^ | 140^a^ | 276^a^ |  | 213 | 0.001 | 0.003 | 0.002 |
| IL-6 |  | 82.3 | 158.4 | 81.4 | 71.4 | 71.4 | 67.8 |  | 18.4 | 0.015 | 0.203 | 0.061 |
| IL-8 |  | 70.0 | 87.8 | 56.3 | 50.9 | 60.6 | 52.5 |  | 11.3 | 0.067 | 0.877 | 0.466 |
| IL-10 |  | 352^a^ | 816^b^ | 386^a^ | 302^a^ | 282^a^ | 348^a^ |  | 109 | 0.040 | 0.108 | 0.049 |
| IL-12 |  | 1,371 | 954 | 870 | 1,080 | 1,336 | 1,046 |  | 187 | 0.464 | 0.285 | 0.225 |
| IL-18 |  | 1,252^a^ | 2,669^b^ | 1,465^a^ | 1,042^a^ | 875^a^ | 1,086^a^ |  | 289 | 0.006 | 0.099 | 0.011 |
| TNFα |  | 168.4 | 167.7 | 138.0 | 103.5 | 89.0 | 95.8 |  | 30.5 | 0.058 | 0.705 | 0.773 |
| GM-CSF |  | 92.0 | 93.6 | 72.7 | 70.7 | 81.3 | 68.8 |  | 10.1 | 0.097 | 0.608 | 0.782 |

^1^ Farrowing treatment main effects.

^2^ Nursery treatment main effects.

^3^ Farrowing by nursery interaction.

^abc^ Means within a row lacking a common superscript differ, (*P* < 0.05)
