# Supplementary material for: Prebiotic galactooligosaccharide improves piglet growth performance and intestinal health associated with alterations of the hindgut microbiota during the peri-weaning period
Source: J Anim Sci Biotechnol. 2024 Jun 13;15:88. doi: 10.1186/s40104-024-01047-y (PMC11170840; doi:10.1186/s40104-024-01047-y)
Supplement: Supplementary file 3 — Additional file 3: Additional Fig. 1. Jejunal morphology1 after weaning (D31) in pigs exposed to farrowing treatments (FC, FG–, FG+) followed by phase 1 nursery diets without (NG–) or with GOS( NG+). [file 40104_2024_1047_MOESM3_ESM.docx]

Additional Fig 1. Jejunal morphology^1^ after weaning (D31) in pigs exposed to farrowing treatments (FC, FG-, FG+) followed by phase 1 nursery diets without (NG-) or with GOS( NG+).

^1^ Pictures were taken at 40X magnification.
